# A sensitized genetic screen to identify regulators of *Caenorhabditis elegans* germline stem cells

**DOI:** 10.1093/g3journal/jkab439

**Published:** 2021-12-23

**Authors:** Sarah Robinson-Thiewes, Aaron M Kershner, Heaji Shin, Kimberly A Haupt, Peggy Kroll-Connor, Judith Kimble

**Affiliations:** 1 Department of Genetics, University of Wisconsin-Madison, Madison, WI 53706, USA; 2 Howard Hughes Medical Institute, Chevy Chase, MD 20815, USA; 3 Department of Biochemistry, University of Wisconsin-Madison, Madison, WI 53706, USA

**Keywords:** Caenorhabditis elegans, stem cells, Notch, DNA polymerase, forward genetics screen

## Abstract

GLP-1/Notch signaling and a downstream RNA regulatory network maintain germline stem cells in *Caenorhabditis elegans*. In mutants lacking the GLP-1 receptor, all germline stem cells enter the meiotic cell cycle precociously and differentiate into sperm. This dramatic germline stem cell defect is called the “Glp” phenotype. The *lst-1* and *sygl-1* genes are direct targets of Notch transcriptional activation and functionally redundant. Whereas single *lst-1* and *sygl-1* mutants are fertile, *lst-1 sygl-1* double mutants are sterile with a Glp phenotype. We set out to identify genes that function redundantly with either *lst-1* or *sygl-1* to maintain germline stem cells. To this end, we conducted forward genetic screens for mutants with a Glp phenotype in genetic backgrounds lacking functional copies of either *lst-1* or *sygl-1.* The screens generated 9 *glp-1* alleles, 2 *lst-1* alleles, and 1 allele of *pole-1*, which encodes the catalytic subunit of DNA polymerase ε. Three *glp-1* alleles reside in Ankyrin repeats not previously mutated. *pole-1* single mutants have a low penetrance Glp phenotype that is enhanced by loss of *sygl-1.* Thus, the screen uncovered 1 locus that interacts genetically with *sygl-1* and generated useful mutations for further studies of germline stem cell regulation.

## Introduction

Stem cells maintain a robust balance between self-renewal and differentiation to ensure tissue homeostasis despite physiological and environmental challenges. Failure to maintain that balance can lead to tissue dysfunction, disease, and death (Simons and Clevers 2011). Therefore, understanding the molecular circuitry governing stem cell regulation is critical. Yet biologically robust regulatory circuits are notoriously difficult to disentangle. 

**Fig. 1.**
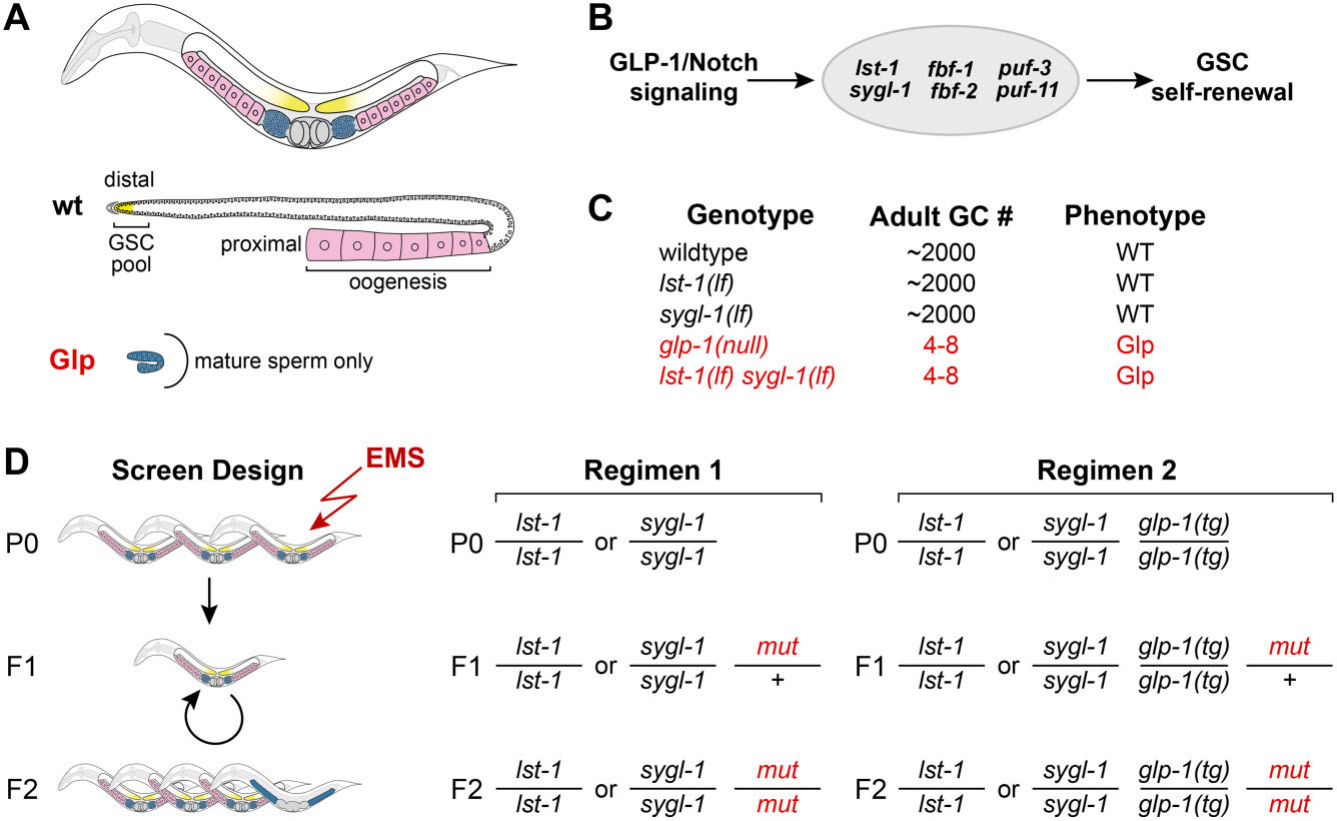
Genetic screens for synthetic Glp mutants. a) Top, adult hermaphrodite has 2-U-shaped gonadal arms (GSCs, yellow; blue, sperm; pink, oocytes). Sperm made during larval development are stored in spermatheca. Middle, wildtype germline with a GSC pool (yellow) distally and oocytes (pink) proximally. Bottom, Glp adult germline with only a few mature sperm (blue). b) Molecular regulation of GSC self-renewal. GLP-1/Notch signaling activates transcription of *lst-1* and *sygl-1*, which are components of the PUF regulatory hub, along with *fbf-1, fbf-2, puf-3*, and *puf-11* (Haupt et al. 2020). c) Adult germ cell (GC) numbers and phenotypes of specified genotypes. d) Strategies to identify genes that have a synthetic Glp phenotype with *lst-1* or *sygl-1*. Regimen 1 mutagenizes *lst-1(lf)* or *sygl-1(lf)* homozygotes and scores for Glp sterility in the F_2_. Regimen 2 mutagenizes *lst-1(lf)* or *sygl-1(lf)* homozygotes that also carry a wildtype *glp-1* transgene, *glp-1(tg)*, to avoid isolation of *glp-1* mutations.

The *C. elegans* germline is a powerful system for the study of stem cell regulation (Hubbard and Schedl 2019). The adult hermaphrodite germline is contained in 2 U-shaped gonadal arms and produces oocytes; sperm are made during larval development and stored for later fertilization (Fig. 1a, top). Germline stem cells (GSCs) are maintained at the distal end of each gonadal arm by a single-celled somatic niche, while GSC daughters differentiate as they move proximally away from the niche and ultimately undergo oogenesis (Fig. 1a, middle) (Hubbard and Greenstein 2000).

GSC self-renewal depends on GLP-1/Notch signaling from the niche and on a downstream RNA regulatory network. In *glp-1* null mutants, GSCs fail to self-renew and instead differentiate precociously into sperm—the “Glp” phenotype (Austin and Kimble 1987) (Fig. 1a, bottom). Downstream of GLP-1/Notch, a “PUF hub” is required for self-renewal (Fig. 1b). This regulatory hub comprises 4 genes encoding PUF RNA-binding proteins (FBF-1, FBF-2, PUF-3, and PUF-11) as well as 2 direct GLP-1/Notch target genes, *lst-1* and *sygl*-1, that encode novel PUF interacting proteins (Crittenden et al. 2002; Kershner et al. 2014; Shin et al. 2017; Haupt et al. 2019; Haupt et al. 2020; Qiu et al. 2019).

The PUF hub is characterized by pervasive genetic redundancy. For example, mutants lacking 3 PUF homologs are able to sustain some GSC self-renewing divisions, but animals lacking all 4 homologs phenocopy *glp-1* null mutants (Haupt et al. 2020). Moreover, single mutants lacking *lst-1* or *sygl-1* are fertile and similar to the wildtype, while *lst-1 sygl-1* double mutants phenocopy *glp-1* null mutants (Fig. 1c) (Kershner et al. 2014). The highly redundant nature of the PUF hub has hampered the identification of its component parts. Indeed, LST-1 and SYGL-1 were not identified using standard forward genetic approaches, but instead were discovered using a candidate gene approach (Kershner et al. 2014), leaving open the possibility that additional components remain unidentified. For example, the LST-1 or SYGL-1 proteins might work with other unknown redundant factors. Here, we describe the results of mutagenesis screens designed to identify regulators that function redundantly with *lst-1* or *sygl-1.*

## Methods

### Strain maintenance

Unless noted otherwise, strains were maintained as previously described (Brenner 1974), at a temperature of 15°C. Balancers used to maintain recovered alleles were *hT2[qIs48]* (Siegfried and Kimble 2002) and *hIn1[unc-54(h1040)]* (Zetka and Rose 1992). Table 1 lists the strains used and their genotypes.

**Table 1.** Strains used in study.

Strain	Genotype	Reference
N2	Wildtype	Brenner (1974)
JK2877	*unc-32(e189) glp-1(q175) III/hT2[qIs48] (I; III)*	This work
JK4356	*lst-1(ok814) I*	Kershner et al. (2014)
JK4774	*lst-1 (ok814) sygl-1 (tm5040) I/hT2[qIs48] (I; III)*	Kershner et al. (2014)
JK4899	*sygl-1(tm5040) I*	Kershner et al. (2014)
JK5135	*sygl-1(tm5040) I; qSi44[Pglp-1::6XMYC::6xHIS::glp-1 3′ end] II*	Sorensen et al. (2020) and this work
JK5203	*lst-1(ok814) I; qSi44[Pglp-1::6MYC::6XHIS::glp-1 3′ end] II*	Sorensen et al. (2020) and this work
JK5209	*lst-1(q827) sygl-1(tm5040) I/hT2[qIs48](I; III)*	This work
JK5277	*lst-1(q826) I/hT2[qIs48](I; III)*	Shin et al. (2017)
JK5305	*lst-1(q827) I/hT2[qIs48](I; III)*	This work
JK5315	*lst-1(q826) sygl-1(tm5040) I/hT2[qIs48] I; III*	Shin et al. (2017)
JK5606	*lst-1(ok814) pole-1(q831) I/hIn1 [unc-54(h1040)] I*	This work
JK5293	*sygl-1(tm5040) pole-1(q831) I/hIn1[unc-54(h1040)] I*	This work
JK5250	*pole-1(q831) I/hIn1[unc-54(h1040)] I*	This work
JK5268	*pole(gk49) I/hIn1[unc-54(1040)] I*	This work
JK5546	*glp-1(q819) III/hT2[qIs48] (I; III)*	This work
JK5547	*glp-1(q824) III/hT2[qIs48] (I; III)*	This work
JK5568	*glp-1(q818) III/hT2[qIs48] (I; III)*	This work
JK5569	*glp-1(q822) III/hT2[qIs48] (I; III)*	This work
JK5570	*glp-1(q825) III/hT2[qIs48] (I; III)*	This work
JK5575	*glp-1(q817) III/hT2[qIs48] (I; III)*	This work
JK5576	*glp-1(q820) III/hT2[qIs48] (I; III)*	This work
JK5577	*glp-1(q821) III/hT2[qIs48] (I; III)*	This work
JK5578	*glp-1(q823) III/hT2[qIs48] (I; III)*	This work

### Screen design and phenotype scoring

We screened for *lst-1* or *sygl-1* enhancers using a modified ethyl methanesulfonate (EMS) protocol (Brenner 1974). Fourth larval stage (L4) hermaphrodites were soaked in 25 mM EMS (Sigma: M0880) for 4 h at room temperature, washed with M9, and placed on plates. F1 progeny were singled onto individual Petri dishes and allowed to self at 15°C. F2 adult progeny were scored for sterility by dissecting scope, and then L4 larvae were scored for a Glp phenotype using a Zeiss Axioskop compound scope equipped with DIC Nomarski optics, as described (Kershner et al. 2014). Each screen was done in 2 ways—first with single mutants *lst-1(ok814)* and *sygl-1(tm5040)* (Fig. 1d, regimen 1) and then with each of the same mutants carrying a transgenic copy of wildtype *glp-1* (Sorensen et al. 2020) in addition to an endogenous copy of wildtype *glp-1* (Fig. 1d, regimen 2).

### Allele identification

Following isolation of a mutant with a Glp phenotype, the starting *lst-1* or *sygl-1* allele was crossed away to test whether the Glp defect depended on loss of *lst-1* or *sygl-1*. Mutations were then mapped to a chromosome and tested for their ability to complement alleles of likely candidate genes. Mutants that were fertile as single mutants and mapped to chromosome I were tested for complementation with *lst-1(ok814) I*. Briefly, the double mutant (e.g. *mut-x sygl-1*) was balanced over the green balancer *hT2[qIs48]*, crossed to *lst-1(ok814) sygl-1(tm5040)/hT2[qIs48]* males, and nongreen L4 male progeny (e.g. *mut-x sygl-1/lst-1 sygl-1*) were scored for the Glp defect. Mutants that were sterile as single mutants and mapped to chromosome III were tested for complementation with the null allele *glp-1(q175) III.* Briefly, *unc-32 glp-1(q175)/hT2[qIs48]* males were mated to each suspected *glp-1* allele and nongreen male progeny scored for the Glp defect. If an allele failed to complement either *lst-1* or *glp-1*, then Sanger sequencing was used to identify the molecular lesion. The *glp-1(q823)* allele was sequenced 2,382 bp upstream of the 5′ UTR and 927 bp downstream of the 3′ UTR in addition to the exons and introns, but no lesion was found.

Whole-genome sequencing was used to identify the likely lesion in *q831*, which was sterile as a single mutant and mapped to the right arm of chromosome I. Briefly, we picked ∼570 adult homozygotes, isolated DNA with Puregene Core Kit A (Qiagen ID: 158667) following the manufacturer’s directions and submitted the DNA (∼100 ng) to the Wisconsin Biotechnology Core for sequencing using an Illumina MiSeq. The genome sequence was uploaded to a Galaxy server and analyzed by CloudMap, as previously described (Minevich et al. 2012). A premature stop codon occurred in 1 gene, *F33H2.5*, which resides on the right arm of chromosome I. *q831* failed to complement *F33H2.5 (gk49)* (*C. elegans* Deletion Mutant Consortium 2012), and the premature stop codon was confirmed by Sanger sequencing of DNA from *q831* homozygotes.

### Assay for temperature sensitivity of glp-1 and pole-1 alleles

Balanced strains carrying *glp-1* or *pole-1* alleles were maintained at 15°C, 20°C, or 25°C for at least 1 generation before homozygous *glp-1* or *pole-1* L4 progeny were scored for a Glp phenotype.

### pole-1 phenotype assay

Homozygous *pole-1 (q831* or *gk49)* animals were distinguished from the balancer *hIn1[unc-54(h1040)]* by their kinked, uncoordinated movement. Homozygous mid-L4 hermaphrodites were raised at 20°C, anesthetized in levamisole, mounted on an agarose pad, and examined using a Zeiss Axioskop compound scope (Crittenden et al. 2017). Vulva formation—wildtype, multivulva, or vulvaless—was scored in addition to germline defects.

### Immunostaining

Strains were maintained at 20°C for immunostaining following published procedure (Crittenden et al. 2017). The SP56 polyclonal antisperm antibody (Ward et al. 1986), a gift from Susan Strome (UCSC, CA, USA), was diluted 1:200. The secondary antibody Alexa Fluor 555 donkey α-mouse (1:1,000, Invitrogen number A31570) was added with DAPI (1 µg/ml) to mark DNA. Gonads were mounted in Vectashield (Vector Laboratories number H-1000), sealed with nail polish, and kept in the dark at 4°C until imaging.

### Microscopy

DAPI/SP56 stained gonads were imaged with a Zeiss Axioskop compound microscope equipped with a Hamamatsu ORCA-Flash4.0 cMos camera and a 63/1.4 NA Plan Apochromat oil immersion objective. Carl Zeiss filter sets 49 and 43HE were used for the visualization of DAPI and Alexa 555. Images were captured using Micromanager (Edelstein et al. 2010, 2014).

### GLP-1 protein conservation

Protein sequences for *C. elegans glp-1* orthologs from other *Caenorhabditis* species were acquired from Wormbase. Sequences of the Ankyrin (ANK) repeats were aligned using M-Coffee to examine amino acid conservation (http://tcoffee.crg.cat/apps/tcoffee/do:mcoffee, last accessed: 7/28/2021) (Notredame et al. 2000).

## Results and discussion

### Screens for Glp mutants in lst-1 and sygl-1 single mutant backgrounds

To identify new GSC regulators and perhaps new components of the PUF hub, we conducted genetic screens for mutations that cause a Glp phenotype in a *lst-1(lf)* or *sygl-1(lf)* single mutant background (Fig. 1d)*.* Our initial screens simply mutagenized *lst-1(lf)* and *sygl-1(lf)* single mutants and scored their F2 progeny for the Glp phenotype (Fig. 1d, regimen 1). We screened 8,749 haploid genomes after mutagenesis of *lst-1(lf)* and 5,504 haploid genomes after mutagenesis of *sygl-1(lf)* (Table 2). This first set of screens recovered 10 mutants. However, outcrossing revealed that all 10 mutants generated animals with a Glp phenotype after *lst-1(lf)* or *sygl-1(lf)* was removed*.* Nine mutations, alleles *q817-q825*, caused a fully penetrant Glp phenotype and mapped to chromosome III (Table 3). Because the *glp-1* locus is large (∼7.4 kb) and located on chromosome III, these 9 mutations were likely *glp-1* alleles. Indeed, all 9 failed to complement *glp-1(null)* (Table 3)*.* The 10th allele *q831* caused a low penetrance Glp phenotype and was mapped to the right arm of chromosome I, at some distance from both *sygl-1* and *lst-1* loci. Therefore, this mutation must be a lesion in some other gene; its identity is described below.

**Table 2.** Summary of screens and alleles recovered.

Parental genotype*^a^*	Copies of *glp-1(+)^b^*	Number of haploid genomes screened	Glp mutants recovered*^c^*	Gene identities	Allele identities
*lst-1(lf) I*	2	8,749	6	6 *glp-1*	*q817*, *q818*, *q819 q820*, *q821*, *q822*
*lst-1(lf) I; qSi44 II*	4	7,922	0	n/a	n/a
*sygl-1(lf) I*	2	5,504	4	3 *glp-1* 1 *pole-1*	*q823*, *q824*, *q825*, *q831*
*sygl-1(lf) I; qSi44 II*	4	3,868	2	2 *lst-1*	*q826*, *q827*

a Alleles were *lst-1(ok814)* and *sygl-1(tm5040)*.

b Animals without qSi44 have 2 endogenous copies of *glp-1(+).* Animals with qSi44 have 2 endogenous and 2 transgenic copies of *glp-1(+).*

c Mutants with Glp phenotype—small germline and sperm to distal end (Austin and Kimble 1987)

**Table 3.** Genetic characterization of sterile mutants from screens.

Allele	LG*^a^*	Glp	Failure to complement*^b^*
*q817*	III	+++	*glp-1(q175)*
*q818*	III	+++	*glp-1(q175)*
*q819*	III	+++	*glp-1(q175)*
*q820*	III	+++	*glp-1(q175)*
*q821*	III	+++	*glp-1(q175)*
*q822*	III	+++	*glp-1(q175)*
*q823*	III	+++	*glp-1(q175)*
*q824*	III	+++	*glp-1(q175)*
*q825*	III	+++	*glp-1(q175)*
*q826*	I	–	*lst-1(ok814)*
*q827*	I	–	*lst-1(ok814)*
*q831*	I	+	*pole-1(gk49)*

+++, 100% penetrance; +, ∼30% penetrance; –, not Glp as single mutants.

a LG, linkage group.

b Allele used in complementation test.

The initial screens were heavily biased for the recovery of *glp-1* alleles. To limit the isolation of more *glp-1* alleles, we introduced a transgenic copy of wildtype *glp-1* into the *lst-1(lf)* and *sygl-1(lf)* single mutants (Fig. 1d, regimen 2; Table 2). The *glp-1* transgene, *qSi44* or *glp-1(tg)*, is a single copy insertion of wildtype *glp-1* on chromosome II that rescues a *glp-1* null mutant (Sorensen et al. 2020). Using the same EMS mutagenesis procedure as before, we screened 7,922 *lst-1(lf); glp-1(tg)* haploid genomes and 3,868 *sygl-1(lf); glp-1(tg)* haploid genomes. No mutants with a Glp phenotype were isolated from *lst-1(lf); glp-1(tg)* but 2 were recovered from *sygl-1(lf); glp-1(tg)* (Table 2). These mutations were subsequently determined to be alleles of *lst-1* (see below). Table 3 summarizes the genetic characterization of alleles recovered from the screen, and Table 4 summarizes their molecular lesions. Our failure to recover *sygl-1* alleles in the *lst-1(lf)* background shows that our screens were not performed to saturation. However, we note that the *sygl-1* locus is relatively small (621 bp coding region) and therefore likely a poor mutagenesis target.

**Table 4.** Molecular lesions in alleles recovered from the screen.

Gene(allele)	Type of mutation	Nucleotide change	Codon change	Amino acid change
*glp-1(q817)*	Missense	C → T	CCG → UCG	P1111S
*glp-1(q818)*	Nonsense	C → T	CAA → UAA	Q98Stop
*glp-1(q819)*	Missense	C → T	CAU → UAU	H1000Y
*glp-1(q820)*	Missense	T → A	AAU → AAA	N992K
*glp-1(q821)*	Nonsense	C → T	CGA → UGA	R499Stop
*glp-1(q822)*	Nonsense	T → G	UAU → UAG	Y176Stop
*glp-1(q823)^a^*	Unknown	Not found	n/a	n/a
*glp-1(q824)*	Substitution	AC → CA in intron 4*^b^*	n/a	n/a
*glp-1(q825)*	Splice site	G → A	n/a	n/a
*lst-1(q826)*	Nonsense	C → T	CGA → UGA	R114Stop
*lst-1(q827)*	Splice site	G → A	n/a	n/a
*pole-1(q831)*	Nonsense	G → A	UGG → UGA	W1899Stop

n/a, not applicable.

a See *Methods* for more details.

b 184^ ^bp from 5′ splice site.

**Table 5.** *glp-1* alleles and temperature sensitivity.

Allele	% Glp 25°C	% Glp 20°C	% Glp 15°C	*n*
N2	0	0	0	20
*q175*	100	100	100	20
*q817*	100	100	100	40*^a^*
*q818*	100	100	100	20
*q819*	100	100	100	40
*q820*	100	100	100	40
*q821*	100	100	100	20
*q822*	100	100	100	20
*q823*	100	100	100	20
*q824*	100	100	100	20
*q825*	100	100	100	20

*n*, number germlines scored.

a For *g817* at 15°C, 38 germlines scored.

### Characterization of lst-1 alleles

**Fig. 2.**
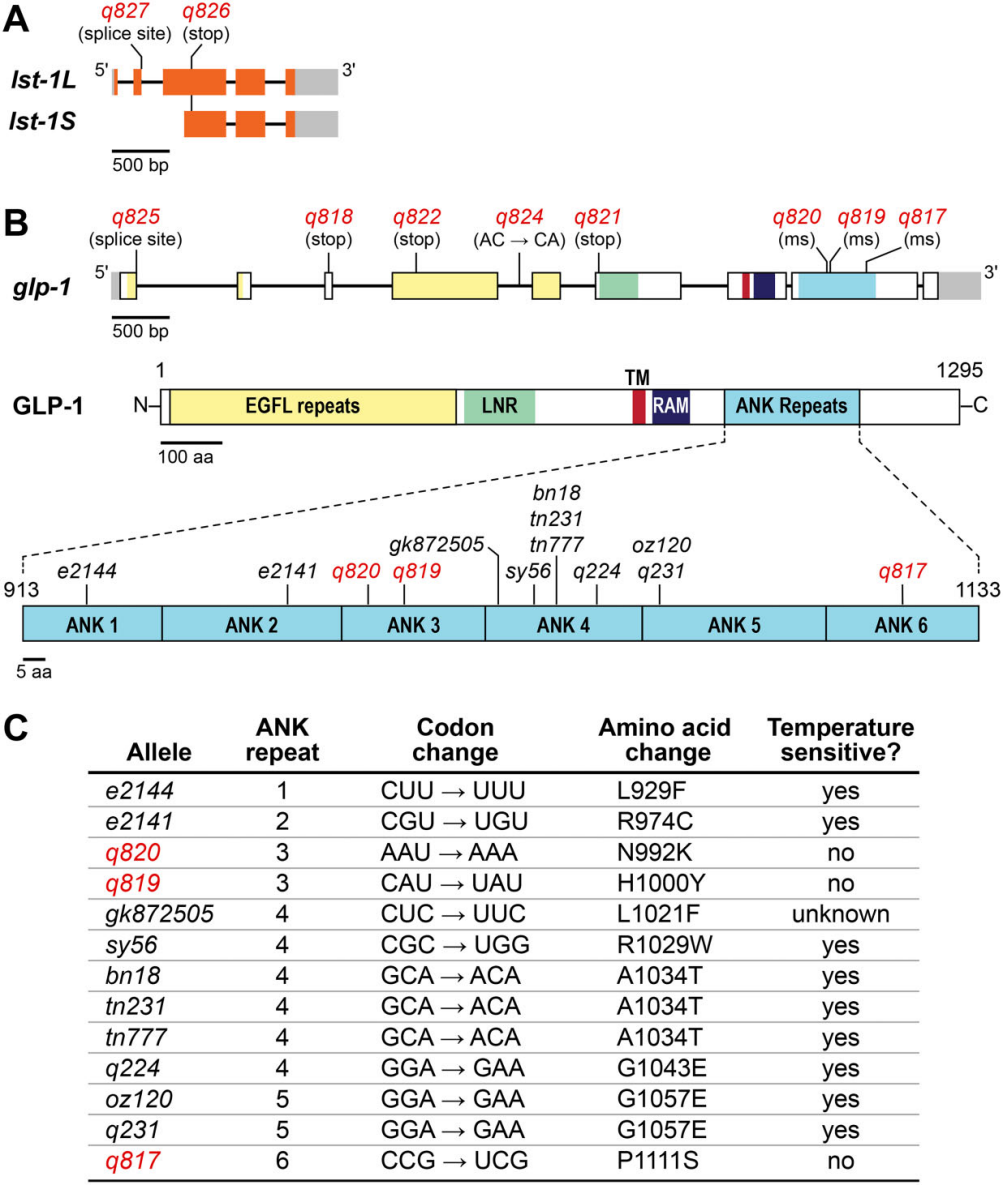
*lst-1* and *glp-1* alleles recovered from screens. Architecture of *lst-1* and *glp-1* loci. Boxes, exons with untranslated regions in gray; introns, lines connecting exons. a) The *lst-1* locus generates 2 RNA isoforms, *lst-1L* and *lst-1S.* Mutations isolated in screens shown above; see Table 4 for molecular changes. b) The *glp-1* locus generates 1 RNA isoform and 1 protein product. Regions within exons are colored according to protein domains: yellow, EGF-like (EGFL) repeats; green, *lin-12/Notch* Repeats (LNR); red, transmembrane domain ; dark blue, RAM domain; light blue, ANK repeats. Mutations in the ANK repeats that are shown below include those from this work (red) and those published previously (Austin and Kimble 1987; Kodoyianni et al. 1992; Berry et al. 1997; Dalfo et al. 2010; Thompson et al. 2013). Not shown are ANK repeat mutations isolated as intragenic suppressors of *glp-1(q231)* and *glp-1(q224)* (Lissemore et al. 1993). Ms, missense. c) Key features of *glp-1* mutations in ANK repeats. See Table 4 for molecular changes in other *glp-1* alleles and Table 5 for temperature sensitivity data.

The *lst-1* locus generates 2 RNA isoforms—1 longer, called *lst-1L*, and 1 shorter, called *lst-1S* (Fig. 2a;Table 4). Most *lst-1* alleles available prior to this work were isolated in deletion screens (Kershner et al. 2014) or engineered by CRISPR/Cas9 gene editing (Haupt et al. 2019). In addition, 1 allele from these screens was previously reported, the nonsense mutant *lst-1(q826)* (Shin et al. 2017)*.* Here, we report a second allele obtained in the screen, *lst-1(q827)*, which alters the 5′ splice site in *lst-1L* intron 2 (Fig. 2a;Table 4). As previously reported for *lst-1(q826), lst-1(q827)* was confirmed by complementation tests and Sanger sequencing. Both alleles are phenotypically similar to previously characterized *lst-1(lf)* mutants: as a single mutant, they appear wildtype (*n* > 50) and as *lst-1 sygl-1* double mutants they were all sterile (*n* > 50) and had the Glp phenotype (*n* = 10). These *lst-1* alleles will prove useful in future studies focused on *lst-1* function.

### Characterization of glp-1 alleles

We identified the molecular lesions in the *glp-1* alleles with Sanger sequencing: *q818*, *q821*, and *q822* were nonsense mutants; *q817*, *q819*, and *q820* were missense mutants and *q825* altered a 5′ splice site (Fig. 2b;Table 4). The *q824* allele had a 2 bp change (AC → CA) in intron 4 that did not affect the 5′ or 3′ splice sites or the branch point (Fig. 2b). We failed to determine the lesion in 1 allele, *q823*, despite sequencing all exons and introns plus 2,382 bp upstream of the transcription start site and 927 bp downstream of the 3′ UTR. Nonetheless, the remaining 8 alleles were all previously unreported *glp-1* lesions.

**Fig. 3.**
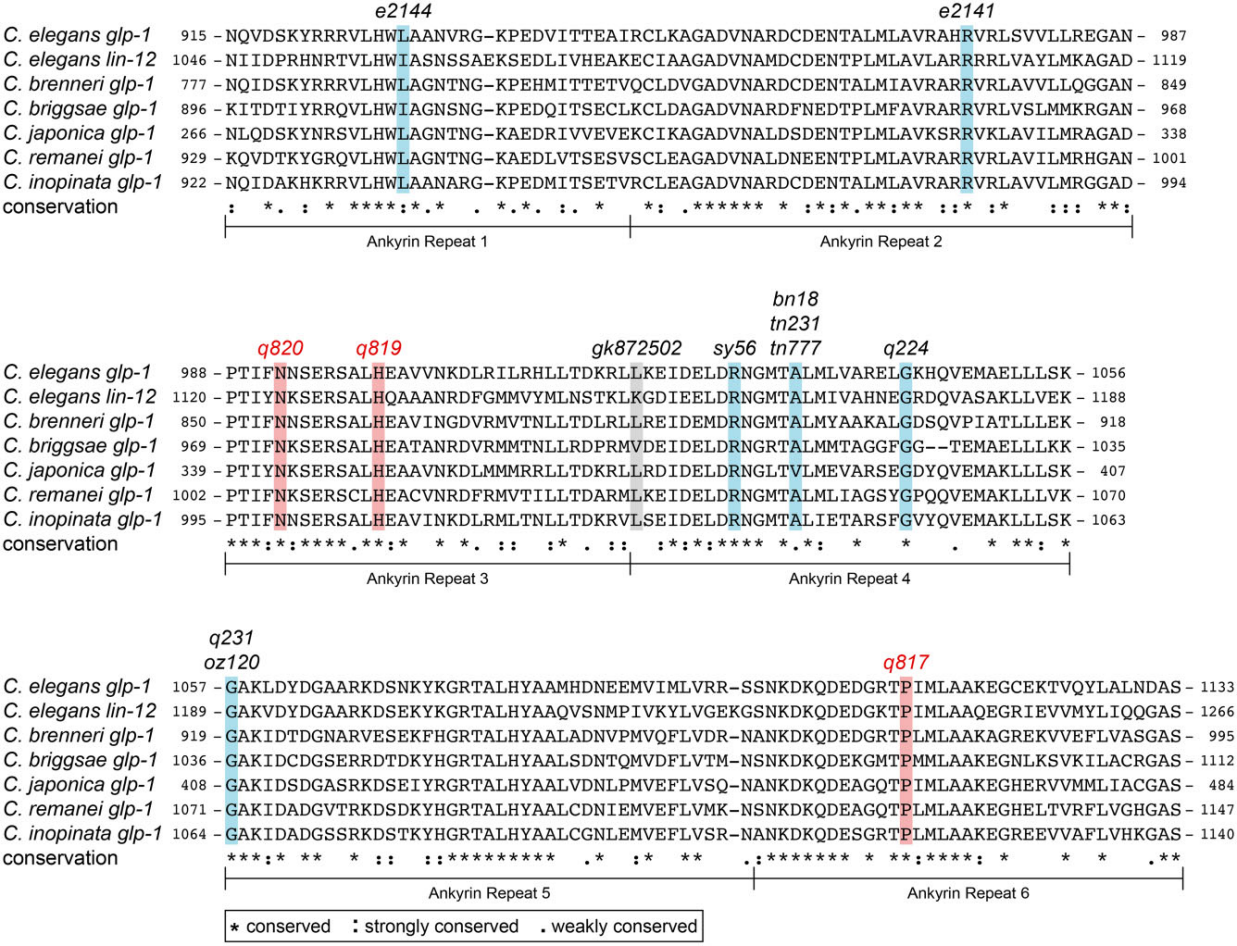
Amino acid alignment for ANK repeats *glp-1* orthologs and in the paralog *lin-12*. Alleles from Fig. 2c are marked. Blue bar, mutation causes sterility at 25°C but not at 15°C; red bar, mutation causes sterility at 15°C, 20°C, and 25°C. The residue affected in *gk872502* is marked by a gray bar, because it has not been tested for temperature sensitivity. ANK repeat location within each paralog is shown beside amino acids. See legend for conservation key.

The 3 *glp-1* missense alleles—*q817*, *q819*, and *q820—*all carry amino acid changes in the ANK repeats (Fig. 2b and c). ANK repeats are conserved across eukaryotes with roles in protein interaction, cell signaling, and disease (Roehl et al. 1996; Mosavi et al. 2004). Many previously identified *glp-1* alleles also have changes in this region. Mutations in ANK repeats 1, 2, 4, and 5 all cause a temperature sensitive Glp phenotype (Kodoyianni et al. 1992; Berry et al. 1997; Nadarajan et al. 2009; Dalfo et al. 2010). Our 3 newly identified missense alleles occur in different repeats, ANK 3 (*q819* and *q820*) and ANK 6 (*q817*) and they are not temperature sensitive (Table 5). All 3 affect conserved residues (Fig. 3). We conclude that the newly identified ANK missense mutations affect residues essential for GLP-1 function. These alleles should prove useful for investigating ANK repeats and their role in Notch signaling.

### Characterization of pole-1(q831)

**Fig. 4.**
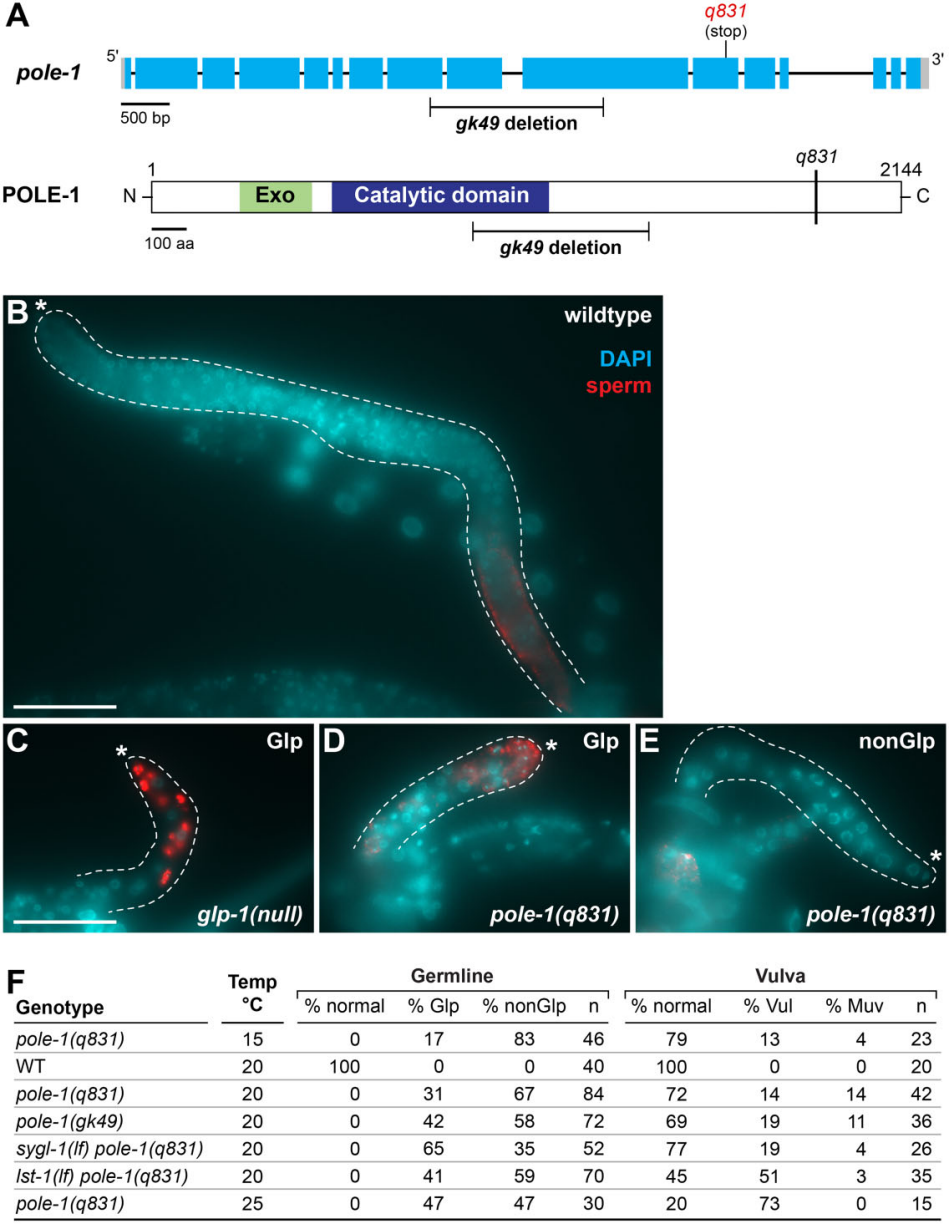
*pole-1* characterization. a) Diagrams of *pole-1* RNA and protein structures*.* Marked mutations include *gk49* (*C. elegans* Deletion Mutant Consortium 2012) and *q831* (this work). Conventions for gene structure as in Fig. 2. Protein domains: exonuclease (Exo) domain, green; DNA polymerase ε catalytic domain, dark blue (Pospiech and Syväoja 2003). Dissected mid-L4 gonads stained with SP56 antibodies for sperm (red) and with DAPI for DNA (blue) (see *Methods*). Dotted line outlines each gonad; asterisk marks the distal end. Scale, 50 µm. b) Wildtype. c) *glp-1* Glp germline. d) Glp *pole-1(q831)* germline. e) NonGlp *pole-1(q831)* germline. f) Low penetrance *pole-1* Glp phenotype is enhanced by loss of *sygl-1*, not *lst-1.* Germline “normal” refers to an adult germline similar to wildtype in size and organization; “Glp” refers to a smaller than normal germline with sperm to distal end; “nonGlp” refers to a smaller than normal germline without sperm at the distal end. Vulva: “normal” refers a vulva similar to a wildtype morphology; “Vul” denotes Vulvaless; “Muv” denotes Multivulva. Temp refers to temperature at which animals were raised (see *Methods*). *n*, number of germlines or vulvas scored.

One mutant allele isolated in the *sygl-1(lf)* background, *q831*, mapped to the right arm chromosome I. Whole-genome sequencing revealed a nonsense mutation R1899Stop in *F33H2.5* (Table 4), which encodes a *C. elegans* ortholog of the catalytic subunit of DNA polymerase ε (Fig. 4a). We confirmed *q831* as an allele of *F33H2.5* by Sanger sequencing, and by its failure to complement *gk49*, a deletion allele in *F33H2.5* that had been generated by the *C. elegans* Knockout Consortium (*C. elegans* Deletion Mutant Consortium 2012)*. F33H2.5* has been named *pole-1* for its DNA polymerase ε orthology.

The *pole-1(q831)* mutation was isolated because *sygl-1(lf) pole-1(q831)* double mutants had a Glp phenotype. During outcrossing, we found that *pole-1(q831)* single mutants were 100% sterile (Fig. 4d–f). To ask if *pole-1* sterility was due to a Glp defect, we examined L4 larvae under DIC/Normaski and also stained dissected gonads with a sperm-specific antibody (SP56) (Ward et al. 1986) and DAPI (Fig. 4b–f) (see *Methods*). Wildtype L4 gonads contain several hundred germ cells, with undifferentiated cells at the distal end and differentiated sperm at the proximal end (Fig. 4b). *glp-1(null)* L4 gonads, in contrast, contain only a few germ cells, all of which have differentiated into SP56-positive sperm extending to the distal end (Fig. 4c). Similar to *glp-1(null)* gonads, the *pole-1(q831)* gonads were physically smaller than wildtype; however, only ∼30% had differentiated sperm extending to the distal end and thus were Glp (Fig. 4d and f). The other ∼70% did not have sperm extending to the distal end and were designated nonGlp steriles (Fig. 4e and f). We also observed a low penetrance Glp phenotype in the deletion strain *pole-1(gk49)* (Fig. 4a and f). Because the Glp phenotype was not fully penetrant at 20°C, we examined *pole-1(q831)* animals raised at 15°C and 25°C. Indeed, the Glp penetrance increased with the temperature—indicating that the Glp defect is temperature sensitive (Fig. 4f). In addition to germline defects, *pole-1* mutants had a range of other defects, consistent with a broad role in development. For example, *pole-1* mutants had vulval defects (Fig. 4f) and were uncoordinated. DNA polymerase ε *pole-1* was not previously been recognized critical for GSC maintenance, though other components of the DNA replication machinery have been implicated in germ cell proliferation (Yoon et al. 2018).

We next asked if the *pole-1* Glp phenotype was enhanced by loss of *lst-1* or *sygl-1.* We found that *pole-1(q831)* single mutants were 30% Glp; *lst-1(lf) pole-1(q831)* double mutants were 41% Glp; and *sygl-1(lf) pole-1(q831)* double mutants were 65% Glp (Fig. 4f). Thus, loss of *sygl-1* is a clear enhancement of the *pole-1* Glp defect, but loss of *lst-1* had a more minor increase and is not clearly an enhancement. Finally, *pole-1* vulval defects were not enhanced (Fig. 4f). We conclude that *sygl-1* is an enhancer of the *pole-1* germline defect.

## Conclusions and future directions

The goal of the mutant screens in *lst-1* and *sygl-1* mutant backgrounds was to identify new regulators of GSC self-renewal. In particular, we sought to test the idea that the LST-1 and SYGL-1 proteins might work with other factors that were similarly redundant. The screens identified 9 alleles of *glp-1*, 2 alleles of *lst-1*, and 1 allele of *pole-1—*the *C. elegans* ortholog of DNA polymerase ε*.* Although the screens were not saturated, identification of *pole-1* with a low penetrance Glp phenotype demonstrates that additional genes likely await discovery. Any additional screens in *lst-1* or *sygl-1* null backgrounds should focus on the modified design with transgenic *glp-1* to avoid isolation of more *glp-1* alleles*.* Alternatively, overexpression of either *lst-1* or *sygl-1* causes a germline tumor (Shin et al. 2017) and so one might seek suppressors of those tumors or enhancers of the low penetrance *pole-1* Glp phenotype.

## Data availability

Strains are available upon request. The authors affirm that all data necessary for confirming the conclusions of the article are present within the article, figures, and tables.
